# Editorial: Exploring female intuition: insights into gendered information processing

**DOI:** 10.3389/fsoc.2025.1750192

**Published:** 2025-12-17

**Authors:** Lois Isenman, Marta Sinclair

**Affiliations:** 1 Brandeis University, Waltham, MA, United States; 2 Griffith University, Brisbane, QLD, Australia

**Keywords:** intuition, women's intuition, unconscious information processing, intuition and attunement, non-local intuition, gender and emotion, gender and bodily experience, women in management and leadership

Intuition is increasingly recognized as essential to navigating the world, especially the volatile environment in which we nowadays live. Constructed largely below awareness, it is often fast, employing pattern recognition to cut through volumes of data (Churchland, 1992; Klein, 1998). Yet it can be slow (Isenman, 2018; Sinclair, 2010), gradually building, in response to often subtle tensions and ambiguities, a network of connections below awareness that eventually lead to deeper understanding (Isenman, 2018). Irrespective of its pace, intuition is becoming crucial for effective functioning in business (Huang, 2025; Sadler-Smith, 2023) and society (Goldberg, 1983).

The aim of this Research Topic is to explore gender-based information processing and its impact on how intuition is used to make decisions, solve problems, and foresee future challenges. Although intuition is increasingly studied, possible differences in women's and men's intuition have been largely ignored in academia, in part taking a back seat to women's drive for equality. In view of the progress here we have chosen to re-visit the enduring notion of women's intuition and examine its validity.

Research shows that men prefer to focus on the most salient or seemingly most important information, whereas women tend to consider and elaborate all the data, including its inherent interrelationships, context, and subtle ambiguities (for a review, see Meyers-Levy and Loken, 2015). Women are also more likely than men to integrate cognitive messages with emotional (Meyers-Levy and Zhu, 2010) and bodily feelings (Alfano et al., 2023). This epistemic carefulness, an implicit kind of caring, mirrors the priority women give to relationships and attunement with others, while men are somewhat more likely to be motivated by efficiency and a sense of agency (Brody and Hall, 2008).

The articles explore women's intuition from multiple perspectives: behavioral economics, quantum physics, evolutionary and cognitive science. All authors agree that there is a special kind of intuition, sometimes fast and sometimes slow, that is inherent (but not exclusive) to women. The commonalities highlight an emphasis on relationship and interconnectivity, the importance of emotional and bodily input, and the role of context. There is agreement that women's intuition can be shaped by both biological and sociocultural factors. While all articles explore women's intuition in the personal sphere, some also highlight how it would enhance management and leadership.

In his integrative review of the gender-based economic and leadership literature, Gómez Tobías posits—without ruling out biological disposition—that cultural and institutional disparities can account for women's tendency toward a relationally-focused, morally aligned intuition. Female intuition is particularly important in conditions of ambiguity and uncertainty, he argues, where the rational and data driven ideals of traditional science and management work poorly. His review shows that women's intuition leads to superior economic performance, and leadership, especially in times of upheaval, in part because it recalibrates attitude to risk. The author proposes that women's ability to sense latent tensions and to integrate emotional, somatic, and contextual factors allows them to anticipate future trends.

Although all articles concur that biology is not destiny, most argue that biology nonetheless gives it a strong push. Verma and Kind postulate that evolutionary forces have forged women's intuition to support infant, and thus species, survival. They point out, for example, that hormonal changes during pregnancy and early post-partum alter perception, making mothers more attuned to their child's needs. The authors argue that the female ability to attune, which they place at core of female intuition, is shaped by entwined biological and environmental influences. Attunement supports neural synchrony, or neural mirroring, between mother and child—although it can also occur between others. Trauma, in contrast, restricts intuition by deregulating the nervous system, thus limiting attunement. Yet trauma victims, often women, can learn to reset their nervous system and restore, and sometimes even enhance intuition. The authors suggest that attunement can be developed by enhancing somatic awareness and tuning into the nervous system.

Similarly, Bradley and Torris focus on the biological (and quantum-physical) underpinnings of women's intuition based on their child-bearing capacity. In agreement with Gómez Tobías, they conclude that women have a special capacity to anticipate emotion-laden events. Yet the enigmatic data and case studies they present suggest non-local intuition—“the body's perception of tacit information from remote objects or events that have yet to happen.” This presentiment is often associated with loved ones, and the heart receives it before the brain. The authors propose that beginning at conception, multiscale quantum entanglement is driven by a mother's unconditional love for her offspring and sustained *in utero* by her heartbeat—the strongest rhythmic activity of the body. It forges harmonic resonance between them and creates a bond of inseparability, which by adolescence develops into a two-way intuitive channel. Similar entanglement can also be established between others.

Likewise, Isenman and Sinclair place receptivity at the core of women's intuition, attributing it to women's traditional childbearing and nurturing role. They emphasize that female receptivity, or attunement, is epistemic as well as emotional. The authors posit that women do more of their higher-level thinking below awareness than men because their comprehensive and relational, or interconnected, way of knowing fits well with intuitive cognition, which depends largely on the interconnections between different pieces and types of information formed beneath consciousness. The extensive gathering of cues can make some intuitions slow, as multiple levels of interconnections form and reconfigure over time. Nevertheless, under stress, as Gómez Tobías also points out, female intuition can function more quickly than usual. The authors propose a bi-directional intuitive channel involving the insula where emotional and bodily, or somatic, feelings can influence cognitive aspects of intuition, and a reverse mode, often used by women, where unconscious cognitive and emotional knowledge is expressed through bodily feelings. They also highlight the ability of women to acknowledge *not knowing*, which allows them to turn more fully toward the potentially complex and far-reaching insight of the unconscious.

Taken together, the articles suggest that women's intuition is characterized by sensitivity to interrelationships, context, latent tensions and anomalous information, as well as to emotional and bodily experience—especially relating to the heart. It allows women to attune to the needs of others and to the subtleties of a given situation somewhat more than men, as well as to anticipate future events. This kind of intuition, which is less recognized in management and leadership, may be especially useful under the conditions of disruption and upheaval in which we increasingly live.
